# Patient journey to Fabry disease diagnosis in the United States: an observational retrospective analysis of two United States claims databases

**DOI:** 10.1186/s13023-025-04041-3

**Published:** 2025-11-27

**Authors:** Alexandra Dumitriu, Gandarvaka Miles, Ana Crespo, Irina Maksimova, Queeny Ip, Michael Jordan, Claudia Leiras, Tiange Yu, Roberto Araujo, Natalia Petruski-Ivleva

**Affiliations:** 1 Sanofi, 450 Water Street, Cambridge, MA 02141 USA; 2 Komodo Health Inc., San Francisco, CA USA; 3 Genesis Research Group, Hoboken, NJ USA

**Keywords:** Administrative claims, Diagnostic delay, Fabry disease, Healthcare resource utilization, Patient journey, Rare diseases

## Abstract

**Background:**

Fabry disease (FD; OMIM # 301500) is a rare, X-linked lysosomal storage disorder caused by mutations in the α-galactosidase A (*GLA*) gene. Deficiency or absence of alpha-galactosidase A (α-Gal A) enzyme activity leads to the accumulation of glycosphingolipids, specifically globotriaosylceramide (GL-3), in lysosomes leading to various symptoms and signs such as neuropathic pain, gastrointestinal manifestations, renal failure, hypertrophic cardiomyopathy and fibrosis, cardiac rhythm disturbances, heart failure, and stroke. This observational study aimed to describe the journey of patients to FD diagnosis (symptoms, comorbidities, related diagnoses, tests, procedures, and healthcare resource utilization) by assessing data from two US claims databases (Optum Clinformatics^®^ Data Mart [Optum CDM] database and Komodo Research Dataset [Komodo RD]).

**Results:**

The study population consisted of 201 patients from the Optum CDM database and 923 patients from the Komodo RD. The mean (SD) age of patients on the index date was 56.0 (20.0) years in the Optum CDM database and 40.5 (21.6) years in the Komodo RD. In the baseline (two years prior to first observed FD diagnosis or treatment) patients had a high prevalence of cardiovascular (72.6% Optum CDM, 56.4% Komodo RD), neurologic (64.2% Optum CDM, 59.3% Komodo RD), gastrointestinal (46.3% Optum CDM, 51.2% Komodo RD), and mental health (32.8% Optum CDM, 36.5% Komodo RD) clinical symptoms and comorbidities. Females showed a higher prevalence of mental health conditions compared to males across both databases. Neurologic and cardiovascular medications were the most commonly prescribed medication classes across both databases (neurologic: 61.2% in both cohorts; cardiovascular: 60.7% Optum CDM, 50.6% Komodo RD). In terms of provider specialty, a high proportion of patients represented in the Optum CDM cohort had claims from family & preventive medicine (73.1%), radiology (72.1%), and internal medicine (61.2%) during the 2 years prior to FD diagnosis. Per Komodo RD, patients had claims from general practitioners (87.4%), radiologists (63.4%), emergency medicine specialists (57.0%) and anesthesiologists (25.8%).

**Conclusions:**

This study assessed the journey of patients with FD to diagnosis based on two large US claims databases, Optum CDM and Komodo RD. The results highlighted a significant burden of cardiovascular, neurologic, and gastrointestinal symptoms and comorbidities, along with associated medication use, in the two years prior to diagnosis. Identifying these comorbidity profiles in patients with FD could shorten time to diagnosis and provide improved disease management.

**Supplementary Information:**

The online version contains supplementary material available at 10.1186/s13023-025-04041-3.

## Background

Fabry disease (FD; OMIM #301500) is a rare multisystemic X-linked lysosomal storage disorder caused by mutations in the α-galactosidase A (*GLA*) gene, which encodes the enzyme α-galactosidase A (α-Gal A), leading to its deficiency. Deficiency or absence of α-Gal A activity results in the accumulation of glycosphingolipids, specifically globotriaosylceramide (GL-3), in lysosomes [1]. While FD has traditionally been considered asymptomatic in early childhood, a recent prospective study (MOPPet study) demonstrates that classic patients with FD, particularly males, show consistent symptom onset around 23.4 months of age, with gastrointestinal symptoms and heat intolerance being common early manifestations. This evolving understanding highlights the importance of early screening and monitoring of at-risk individuals, even in infancy and early childhood [2].

The implementation of newborn screening programs has improved detection of FD, providing more accurate epidemiological data [3–5]. The global prevalence of FD, as determined through clinical assessments, is estimated to be between 1 in 40,000 and 1 in 170,000 individuals. In the United States (US), the prevalence is approximately 1 in 21,973 individuals [6].

There are two phenotypes of FD: classic and late-onset. The classic phenotype is observed in patients with severely deficient α-Gal A activity, whereas patients with residual α-Gal A activity develop late-onset phenotype [7, 8]. Patients with the classic phenotype tend to exhibit symptoms earlier in life including peripheral (neuropathic) pain, gastrointestinal manifestations, hypo- or hyperhidrosis, angiokeratomas, and cornea verticillata [8]. If the disease remains untreated, FD gradually progresses, putting these patients at risk of developing progressive renal failure, hypertrophic cardiomyopathy and fibrosis, cardiac rhythm disturbances, heart failure, stroke, and sudden death [9]. Hence, periodic disease monitoring and dynamic assessment of clinical status are essential in FD management. Patients with late-onset FD typically experience symptom onset later in life and exhibit slower disease progression. Nonetheless, organ dysfunction could occur, with the heart being the most commonly affected organ [7]. Signs and symptoms of FD are typically more severe and rapidly progressive among hemizygous male patients than among heterozygous female patients [10, 11]. However, the majority of heterozygous female patients present with a wide spectrum of signs and symptoms, such as cardiovascular and neurologic symptoms; these manifestations seem to be associated with skewed X chromosome-inactivation favoring the mutant *GLA* allele [12].

The FDA-approved therapies for FD in the US include: (1) enzyme replacement therapies (agalsidase-beta [approved in 2003] and pegunigalsidase-alpha [approved in 2023]) and (2) chaperone therapy, migalastat (approved in 2018), indicated only for patients with specific variants of the *GLA* gene [13–15]. Patients are also administered adjunctive therapies to control renal, cardiac, gastrointestinal and neurologic complications [8]. Although approved therapies for FD are available, patients are frequently misdiagnosed due to a limited awareness within different medical specialties involved in the treatment of patients with FD, as well as the heterogeneity in disease presentation and multisystemic nature of the disease. Thus, delays in initiating disease-modifying therapy are frequent and patients often remain untreated for several years [16–18]. Optimal patient outcomes can only be attained by early diagnosis; careful selection of the most appropriate treatment strategy based on patient’s age, sex, disease genotype and phenotype; and timely initiation of treatment [8].

Understanding the patient journey to diagnosis is crucial for identifying opportunities for earlier detection and intervention. The objective of this observational study was to describe patients’ journey to FD diagnosis, including symptoms, comorbidities, related diagnoses, tests, procedures, and healthcare resource utilization, by analyzing data from two US claims databases, thereby emphasizing the importance of early disease diagnosis and tailored treatment strategies for optimal patient outcomes. Given that sex differences in disease presentation have been previously documented in the literature, results are presented separately for males and females [3].

## Methods

**Fig. 1:**
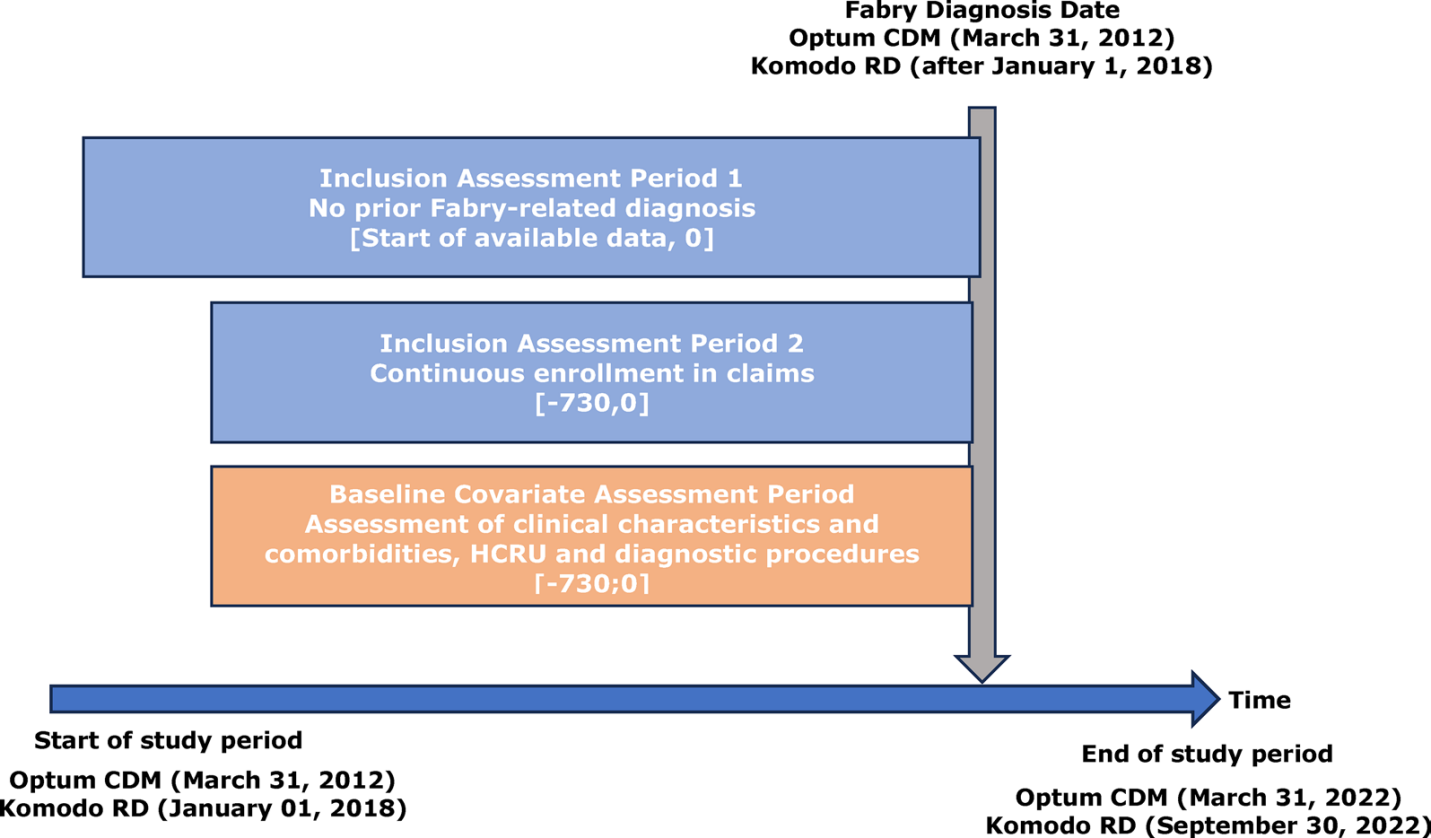
Study design. CDM, Clinformatics^®^ Data Mart; HCRU, healthcare resource utilization; RD, Research Dataset. The index date (day 0) represents the date of first Fabry disease diagnosis. The inclusion assessment periods and baseline covariate assessment period are shown relative to the index date. Study periods: Optum CDM (March 31, 2012–March 31, 2022) and Komodo RD (January 1, 2018–September 30, 2022)

This was an observational retrospective study of patients with FD represented in two US administrative claims databases: Optum Clinformatics^®^ Data Mart (Optum CDM) and Komodo Research Dataset (Komodo RD). Patients were identified from March 31, 2012 to March 31, 2022 in Optum CDM and from January 01, 2018 to September 30, 2022 in Komodo RD (Fig. 1). In each database, patients’ journey to diagnosis, including symptoms, comorbidities, related diagnoses, tests, procedures, and specialty care resource utilization were evaluated. Each patient’s index date was the date of the first observed claim with either International Classification of Diseases-9 (ICD-9) codes for lipidosis, ICD-10 codes for Fabry, or Fabry medication, as defined in the patient selection criteria described below.

### Data sources

#### Optum^®^ CDM

Optum’s de-identified CDM is a database consisting of administrative health claims records for members of large commercial and Medicare Advantage health plans. This dataset is statistically de-identified using the Expert Determination method in compliance with the Health Insurance Portability and Accountability Act (HIPAA) and is managed according to the Optum customer data-use agreements. The Optum CDM administrative claims submitted for payment by providers and pharmacies are verified, adjudicated, and de-identified prior to inclusion. These data, including patient-level enrollment information, were derived from the claims submitted for all medical and pharmacy healthcare services, with information related to healthcare costs and resource utilization. The dataset represents a geographically diverse population across all 50 states of the US.

#### Komodo RD

The Komodo RD is an optimized schema of the underlying Healthcare Map™ from Komodo Health for real-world evidence generation and Health Economics and Outcome Research. It consists of administrative data and claims, which are routinely collected health services utilization records and expenditures for over 330 million de-identified unique individuals in the US. This dataset includes commercially insured patients, as well as those covered by Medicare Advantage and Medicaid plans. In compliance with HIPAA, the privacy-preserving tokens of this dataset offer extended patient-level observations of medical encounters and outpatient pharmacy dispensing via linkage across health and pharmacy insurance plans. The earliest available data are from 2016. Specialty datasets, such as genomics, laboratory test results, and electronic medical records, are readily accessible via additional linkage. For this analysis, only closed claims were utilized.

### Data analysis and patient identification

The Optum CDM and Komodo RD databases were analyzed separately to ensure no duplication of patient records. Due to the de-identified nature of the data and Optum Clinformatics’ licensing specifications, direct patient matching across databases was not possible.

### Patient selection criteria

The selected patients were newly diagnosed with FD and met the following inclusion and exclusion criteria; index date was the day of the first observed evidence for FD, defined using one of the following inclusion criteria:

Inclusion criteria:

- At least two claims with ICD-10 code for FD (E75.21) OR.
- At least one claim with ICD-10 code for FD or ICD-9 code for lipidoses (272.7), followed by a claim for FD treatment (agalsidase beta or migalastat) OR.
- At least one claim with ICD-9 code for lipidoses, followed by ICD-10 code for FD OR.
- At least one claim for FD treatment (agalsidase beta or migalastat), with no prior FD-related claims with ICD-9/10 codes.

All patients were required to have at least 2 years of continuous enrollment in a health plan prior to index date.

Exclusion criteria:

Patients with evidence of FD/lipidoses or FD-related treatment prior to the index were excluded.

### Identification of clinical symptoms and comorbidities

Clinical symptoms and comorbidities were primarily identified using International Classification of Diseases, Ninth Revision (ICD-9) and Tenth Revision (ICD-10) diagnosis codes from medical claims. These codes were used to categorize patients’ conditions into broader groups, such as cardiovascular, neurologic, gastrointestinal, and mental health, among others. This approach allows for a more direct identification of diagnosed conditions, although it is limited by the accuracy and completeness of diagnostic coding in the claims data. Medication use was analyzed separately to provide additional context to the patients’ clinical profiles and treatment patterns.

### Outcome measures

Demographics (age, sex, race/ethnicity, region) and the specialty of diagnosing providers were evaluated on index date. Prevalence of clinical symptoms, comorbidities, and concomitant therapy use prior to diagnosis were assessed during the 2-year baseline and described as overall and separately for year 1 and year 2 prior to index date. Frequency of provider visits, specialty visits, lab tests, procedures, and hospitalizations prior to diagnosis were also described. Comorbidities were grouped into the following categories: cardiovascular, cerebrovascular, gastrointestinal, mental health, metabolic, neurologic, ocular, pulmonary, renal, sweating, and others (angiokeratoma, deafness, hypoacusia and hearing loss, and tinnitus). Count of comorbidity categories and commonly observed combinations of comorbidity categories were evaluated for individuals having > 1 comorbidity during the baseline period.

### Statistical analysis

Continuous variables were summarized using mean, standard deviation (SD), median and interquartile range (IQR). Counts and percentages were calculated for categorical variables. Additionally, analyses were performed separately by baseline year (year 1 of baseline: 365 days to 1 day prior to index date; year 2 of baseline: 730 days to 366 days prior to index date) and results presented in the instances where the difference between the two baseline years was at least 10% points (deemed as meaningful increase over time).

To describe healthcare resource utilization (HCRU) during the baseline period, the number and proportion of patients with at least one event (specialist visits, outpatient visits, emergency room (ER) visits, all-cause hospitalizations, cardiovascular disease (CVD)-related hospitalizations, chronic kidney disease (CKD)-related hospitalizations, and laboratory procedures were calculated. Mean (SD) number of visits per patient per year among those with at least one of the events of interest was estimated. The duration of inpatient hospitalization (in days) was described for patients with at least one inpatient event. Moreover, CVD- or CKD-related hospitalization rates, defined as a hospitalization with CVD- or CKD-related ICD-10 codes, respectively, in the primary position on the inpatient claim, were also described.

All analyses were stratified by sex (male and female). No formal statistical comparisons were performed.

## Results

### Baseline demographics

The FD population in the Optum CDM database consisted of 201 patients and, in the Komodo RD, 923 patients were identified. The mean (SD) age of patients on index date was 56.0 (20) years in the Optum CDM database and 40.5 (21.6) years in the Komodo RD. In the Optum CDM and Komodo RD, 48.3% and 42.0% of patients were male, respectively; distribution of age was similar among male and female patients in both databases. Among patients with known race/ethnicity data, the Optum CDM and Komodo RD cohorts consisted of 69.2% (135/195) and 59.4% (392/660) White patients, respectively. For both databases, the highest proportion of patients resided in the southern region of the US (Optum CDM: 43.8% vs. Komodo RD: 38.5%) (Table 1).

**Table 1:** Characteristics of patients with FD on the index date

Characteristic	Optum CDM			Komodo RD		
	Overall (*N* = 201)	Female (*n* = 104)	Male (*n* = 97)	Overall (*N* = 923)	Female (*n* = 535)	Male (*n* = 388)
Age at diagnosis, mean (SD), median	56.0 (20.0), 59	55.0 (20), 58	56.0 (20.0), 59	40.5 (21.6), 42	40.6 (20.5), 41	40.5 (23.0), 45
*Race/ethnicity, n (%)*						
White	135 (67.2)	72 (69.2)	63 (64.9)	392 (42.5)	229 (42.8)	163 (42.0)
Hispanic	31 (15.4)	15 (14.4)	16 (16.5)	108 (11.7)	61 (11.4)	47 (12.1)
Black	26 (12.9)	14 (13.5)	12 (12.4)	83 (9.0)	47 (8.8)	36 (9.3)
Asian	3 (1.5)	0 (0)	3 (3.1)	33 (3.6)	16 (3.0)	17 (4.4)
Other	–	–	–	44 (4.8)	22 (4.1)	22 (5.7)
Unknown	6 (3.0)	3 (2.9)	3 (3.1)	263 (28.5)	160 (29.9)	103 (26.5)
*Region, n (%)*						
Midwest	59 (29.4)	36 (34.6)	23 (23.7)	314 (34.0)	178 (33.3)	136 (35.1)
North	17 (8.5)	6 (5.8)	11 (11.3)	128 (13.9)	64 (12.0)	64 (16.5)
South	88 (43.8)	47 (45.2)	41 (42.3)	355 (38.5)	214 (40.0)	141 (36.3)
West	37 (18.4)	15 (14.4)	22 (22.7)	126 (13.7)	79 (14.8)	47 (12.1)

The index date represents the date of first evidence of Fabry disease (i.e., FD diagnosis or treatment)

*CDM,* Clinformatics^®^ Data Mart; *RD,* research dataset; *SD* standard deviation

### Diagnosing provider specialty

**Fig. 2:**
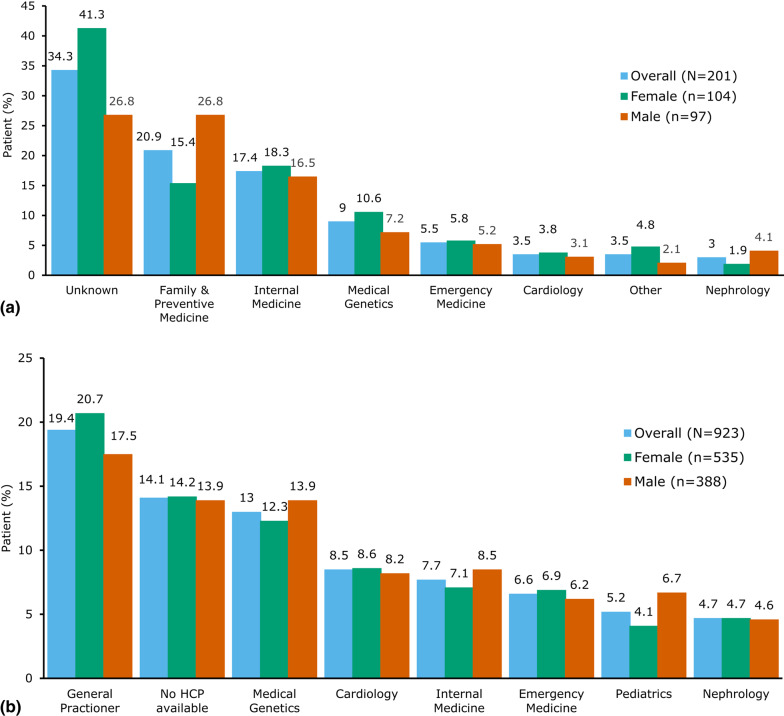
Diagnosing provider specialty for patients with FD in the US.** a** Optum CDM. CDM, Clinformatics^®^ Data Mart; FD, Fabry disease.** b** Komodo RD. HCP, Healthcare practioner; RD, Reseach Dataset

Within the Optum CDM cohort, provider specialty on the first observed claim with FD codes was unknown for 34.3% of patients. Among the known provider specialties, the most common were family & preventive medicine (20.9%), internal medicine (17.4%), and medical genetics (9.0%) (Fig. 2a).

In the Komodo RD, provider specialty on the first observed claim with FD codes was unknown for 3.7% of patients (data not shown). General practitioners (19.4%), medical geneticists (13.0%), cardiologists (8.5%), and internal medicine specialists (7.7%) were the most commonly known diagnosing provider specialists (Fig. 2b).

### Clinical symptoms and comorbidities

#### Optum CDM

In the 2-year baseline period, cardiovascular (72.6%), neurologic (64.2%), gastrointestinal (46.3%), mental health (32.8%), and metabolic (31.8%) clinical symptoms and comorbidities were the most prevalent comorbidity categories observed among patients with FD **(**Table 2**)**. Furthermore, 29.9% of patients had claims for arrhythmia, 14.4% for atrial fibrillation, and 10.9% for bradycardia; 32.3% of patients had claims for abdominal pain, 58.7% for chronic pain, and 27.4% for neuropathic pain. Hypoacusia and hearing loss were prevalent in 9.5% of patients and acute kidney failure in 8.0% (Table 2). Many disease manifestations were more prevalent in female patients than in male patients. Notably, a large sex difference in prevalence of mental health conditions (including depression and anxiety) was observed, with 37.5% and 27.8% of female and male patients receiving one or more related diagnoses, respectively (Table 2).

**Table 2:** Clinical symptoms and comorbidities among patients with FD in the 2 years prior to the index date

Subgroup	Optum CDM			Komodo RD		
	Overall, *N* = 201, *n* (%)	Female, *n* = 104, *n* (%)	Male, *n* = 97, *n* (%)	Overall, *N* = 923, *n* (%)	Female, *n* = 535, *n* (%)	Male, *n* = 388, *n* (%)
**Cardiovascular**	**146 (72.6)**	**76 (73.1)**	**70 (72.2)**	**521 (56.4)**	**287 (53.6)**	**234 (60.3)**
Angina pectoris	48 (23.9)	25 (24.0)	23 (23.7)	135 (14.6)	57 (10.7)	78 (20.1)
Arrhythmia	60 (29.9)	37 (35.6)	23 (23.7)	227 (24.6)	123 (23.0)	104 (26.8)
Atrial fibrillation	29 (14.4)	18 (17.3)	11 (11.3)	80 (8.7)	38 (7.1)	42 (10.8)
Bradycardia	22 (10.9)	13 (12.5)	9 (9.3)	59 (6.4)	32 (6.0)	27 (7.0)
Cardiac arrest or sudden cardiac death	2 (1.0)	2 (1.9)	0 (0)	13 (1.4)	5 (0.9)	8 (2.1)
Cardiomyopathy	21 (10.4)	9 (8.7)	12 (12.4)	73 (7.9)	24 (4.5)	49 (12.6)
Congestive heart failure	34 (16.9)	18 (17.3)	16 (16.5)	87 (9.4)	36 (6.7)	51 (13.1)
Diastolic heart failure	22 (10.9)	12 (11.5)	10 (10.3)	42 (4.6)	19 (3.6)	23 (5.9)
Hyperlipidemia	113 (56.2)	56 (53.8)	57 (58.8)	344 (37.3)	186 (34.8)	158 (40.7)
Hypertension	115 (57.2)	58 (55.8)	57 (58.8)	377 (40.8)	195 (36.4)	182 (46.9)
Myocardial infarction	8 (3.9)	4 (3.8)	4 (4.1)	33 (3.6)	15 (2.8)	18 (4.6)
Non-sustained ventricular tachycardia	6 (3.0)	2 (1.9)	4 (4.1)	34 (3.7)	11 (2.1)	23 (5.9)
Paroxysmal supraventricular tachycardia	5 (2.5)	3 (2.9)	2 (2.1)	36 (3.9)	21 (3.9)	15 (3.9)
Systolic heart failure	20 (9.9)	10 (9.6)	10 (10.3)	54 (5.9)	23 (4.3)	31 (8.0)
**Neurologic**	**129 (64.2)**	**65 (62.5)**	**64 (66.0)**	**547 (59.3)**	**331 (61.9)**	**216 (55.7)**
Acroparesthesia	23 (11.4)	12 (11.5)	11 (11.3)	85 (9.2)	51 (9.5)	34 (8.8)
Chronic pain	118 (58.7)	61 (58.7)	57 (58.8)	508 (55.0)	304 (56.8)	204 (52.6)
Neuropathic pain	55 (27.4)	27 (26.0)	28 (28.9)	264 (28.6)	166 (31.0)	98 (25.3)
Vertigo	11 (5.5)	7 (6.7)	4 (4.1)	24 (2.6)	16 (3.0)	8 (2.1)
**Gastrointestinal**	**93 (46.3)**	**51 (49.0)**	**42 (43.3)**	**473 (51.2)**	**313 (58.5)**	**160 (41.2)**
Abdominal pain	65 (32.3)	37 (35.6)	28 (28.9)	326 (35.3)	238 (44.5)	88 (22.7)
Anorexia	17 (8.5)	9 (8.7)	8 (8.2)	67 (7.3)	37 (6.9)	30 (7.7)
Constipation	34 (16.9)	21 (20.2)	13 (13.4)	130 (14.1)	81 (15.1)	49 (12.6)
Diarrhea	23 (11.4)	12 (11.5)	11 (11.3)	143 (15.5)	93 (17.4)	50 (12.9)
Nausea and vomiting	5 (2.5)	2 (1.9)	3 (3.1)	219 (23.7)	152 (28.4)	67 (17.3)
**Mental health**	**66 (32.8)**	**39 (37.5)**	**27 (27.8)**	**337 (36.5)**	**235 (43.9)**	**102 (26.3)**
Anxiety	49 (24.4)	27 (26.0)	22 (22.7)	280 (30.3)	202 (37.8)	78 (20.1)
Depression	45 (22.4)	29 (27.9)	16 (16.5)	222 (24.1)	158 (29.5)	64 (16.5)
*Metabolic*	64 (31.8)	30 (28.8)	34 (35.1)	156 (16.9)	81 (15.1)	75 (19.3)
Type 2 diabetes	64 (31.8)	30 (28.8)	34 (35.1)	156 (16.9)	81 (15.1)	75 (19.3)
**Renal**	**48 (23.9)**	**24 (23.1)**	**24 (24.7)**	**162 (17.6)**	**81 (15.1)**	**81 (20.9)**
Acute kidney failure	16 (8.0)	11 (10.6)	5 (5.2)	88 (9.5)	40 (7.5)	48 (12.4)
Albuminuria	14 (7.0)	7 (6.7)	7 (7.2)	50 (5.4)	28 (5.2)	22 (5.7)
Chronic kidney disease	38 (18.9)	19 (18.3)	19 (19.5)	27 (2.9)	12 (2.2)	15 (3.9)
CKD Stage 1	2 (1.0)	1 (1.0)	1 (1.0)	13 (1.4)	7 (1.3)	6 (1.5)
CKD Stage 2	5 (2.5)	2 (1.9)	3 (3.1)	30 (3.3)	18 (3.4)	12 (3.1)
CKD Stage 3	20 (10.0)	8 (7.7)	12 (12.4)	64 (6.9)	27 (5.0)	37 (9.5)
CKD Stage 4	1 (0.5)	0 (0)	1 (1.0)	32 (3.5)	13 (2.4)	19 (4.9)
CKD Stage 5 or ESRD	5 (2.5)	4 (3.8)	1 (1.0)	16 (1.7%)	9 (1.7)	7 (1.8)
CKD Stage unspecified	5 (2.5)	4 (3.8)	1 (1.0)	69 (7.5%)	32 (6.0)	37 (9.5)
Proteinuria	16 (8.0)	9 (8.7)	7 (7.2)	53 (5.7%)	29 (5.4)	24 (6.2)
Pulmonary Disease	37 (18.4)	20 (19.2)	17 (17.5)	113 (12.2)	57 (10.7)	56 (14.4)
Chronic bronchitis	2 (1.0)	2 (1.9)	0 (0)	7 (0.8)	3 (0.6)	4 (1.0)
COPD	35 (17.4)	18 (17.3)	17 (17.5)	103 (11.2)	55 (10.3)	48 (12.4)
Emphysema	9 (4.5)	7 (6.7)	2 (2.1)	20 (2.2)	10 (1.9)	10 (2.6)
*Other*	26 (12.9)	15 (14.4)	11 (11.3)	82 (8.9)	44 (8.2)	38 (9.8)
Angiokeratoma	8 (4.0)	3 (2.9)	5 (5.2)	9 (1.0)	4 (0.7)	5 (1.3)
Deafness	6 (3.0)	1 (1.0)	5 (5.2)	–	–	–
Hypoacusia and hearing loss	19 (9.5)	12 (11.5)	7 (7.2)	66 (7.2)	34 (6.4)	32 (8.2)
Tinnitus	6 (3.0)	5 (4.8)	1 (1.0)	24 (2.6)	14 (2.6)	10 (2.6)
**Cerebrovascular**	**19 (9.5)**	**13 (12.5)**	**6 (6.2)**	**86 (9.3)**	**43 (8.0)**	**43 (11.1)**
Seizures	5 (2.5)	4 (3.8)	1 (1.0)	48 (5.2)	25 (4.7)	23 (5.9)
Stroke	11 (5.5)	6 (5.8)	5 (5.2)	44 (4.8)	21 (3.9)	23 (5.9)
TIA	6 (3.0)	4 (3.8)	2 (2.1)	19 (2.1)	10 (1.9)	9 (2.3)
**Ocular**	**18 (9.0)**	**8 (7.7)**	**10 (10.0)**	**18 (2.0)**	**14 (2.6)**	**4 (1.0)**
Cornea verticillata	0 (0)	0 (0)	0 (0)	4 (0.4)	4 (0.7)	–
Subcapsular cataracts	18 (9.0)	8 (7.7)	10 (10.3)	14 (1.5)	10 (1.9)	4 (1.0)
**Sweating abnormalities**	**3 (1.5)**	**2 (1.9)**	**1 (1.0)**	**18 (2.0)**	**9 (1.7)**	**9 (2.3)**
Anhidrosis/hypohidrosis	0 (0)	0 (0)	0 (0)	2 (0.2)	-	2 (0.5)
Hyperhidrosis	3 (1.5)	2 (1.9)	1 (1.0)	16 (1.7)	9 (1.7)	7 (1.8)

The index date represents the date of first evidence of Fabry disease (i.e., FD diagnosis or treatment)

CDM, Clinformatics^®^ Data Mart; CKD, chronic kidney disease; COPD, chronic obstructive pulmonary disease; ESRD, end-stage renal disease; FD, Fabry disease; RD, Research Dataset; TIA, transient ischemic attack

Multimorbidity was also common in this population, with approximately 13.4% of patients with comorbidities in three or more categories, such as a combination of cardiovascular, neurologic, and gastrointestinal or mental health comorbidities. Another 8.0% of patients had two manifestations, including cardiovascular and neurologic (5.5%) or cardiovascular and metabolic (2.5%) comorbidities (data not shown).

The proportion of male patients receiving care for cardiovascular symptoms and conditions increased closer to index date, from 57.7% in year 2 of baseline to 69.1% in year 1 of baseline. No change was observed for female patients. The proportion of female patients with gastrointestinal symptoms increased from 28.8% in year 2 of baseline to 39.4% in year 1 of baseline, whereas no notable change was observed for male patients (data not shown).

#### Komodo RD

In the 2-year baseline period, neurologic (59.3%), cardiovascular (56.4%), gastrointestinal (51.2%), and mental health (36.5%) were the most prevalent comorbidity groups (Table 2). Female patients had lower prevalence of cardiovascular and cerebrovascular symptoms and higher prevalence of neurologic, mental health, and gastrointestinal symptoms than male patients (Table 2).

An increase in the proportion of female patients with gastrointestinal symptoms was observed from year 2 (36.3%) of baseline to year 1 (46.5%) of baseline (data not shown). Approximately 37.0% of patients received diagnoses within at least three comorbidity categories and 24.0% of patients in at least four categories (data not shown). The most commonly observed comorbidity combination was cardiovascular, gastrointestinal, mental health, and neurologic comorbidities (7.6%), followed by cardiovascular, gastrointestinal and neurologic comorbidities (5.6%), and gastrointestinal, mental health, and neurologic comorbidities (4.4%) (data not shown).

Among the symptoms and comorbitidities assessed, pain was one of the most commonly observed in patients with FD: 55.0% of patients had claims for chronic pain, 35.3% for abdominal pain, and 28.6% for neuropathic pain. Nearly 25% of patients had claims for arrhythmia, 8.7% for atrial fibrillation, and 6.4% for bradycardia. 23.7% of patients had claims for for nausea and vomiting, hypoacusia and hearing loss were prevalent in 7.2% of patients and acute kidney failure in 9.5% (Table 2).

### Concomitant therapy use and procedures

#### Optum CDM

**Fig. 3:**
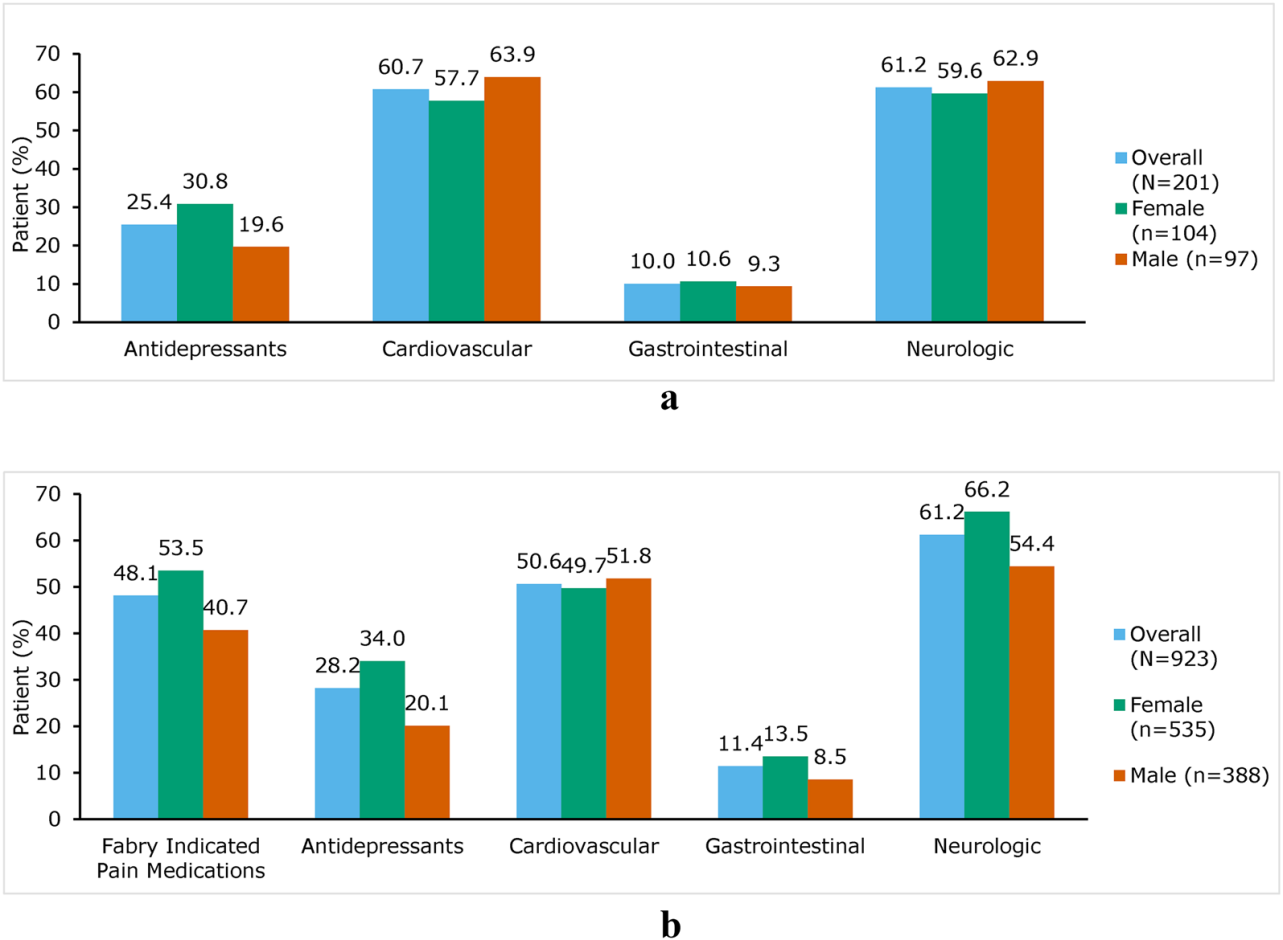
Concomitant therapy use among patients with FD in the 2-year baseline period prior to index date.** a** Optum CDM. CDM, Clinformatics^®^ Data Mart.** b** Komodo RD. RD, Research Dataset

In the overall population, cardiovascular (60.7%) and neurologic medications (61.2%) were the most commonly dispensed medication classes during baseline, followed by antidepressants (25.4%) and gastrointestinal therapy (10.0%) (Fig. 3a). The prevalence of cardiovascular and neurologic therapy use was similar between female and male patients (cardiovascular: 57.7% vs. 63.9%; neurologic: 59.6% vs. 62.9%); however, use of antidepressants was more prevalent among female patients than male patients (30.8% vs. 19.6%, respectively) (Fig. 3a). Cardiovascular procedures were the most commonly observed procedure type in the overall population, with higher proportion of male patients undergoing these procedures than female patients (18.6% vs. 9.6%, respectively). Pacemaker insertion, removal, or repair (7.5%) was the most common type of cardiovascular procedure, followed by catheter ablation (7.0%). Among female patients, 7.7% underwent a pacemaker-related procedure and 2.9% underwent catheter ablation. Among male patients, catheter ablation (11.3%) was the most common procedure, followed by angioplasty (8.2%) (data not shown).

#### Komodo RD

Prior to FD diagnosis, neurologic medications were the most commonly dispensed (61.2%), followed by medications for neuropathic pain (48.1%), and antidepressants (28.2%) (Fig. 3b). Overall, the proportion of patients who underwent cardiovascular or renal procedures was 9.2% and 2.9%, respectively. Most commonly observed cardiovascular procedures were catheter ablation (4.7%) and pacemaker (3.7%). The results for years 1 and 2 of the baseline period were consistent in both databases (data not shown).

### Specialist visits

#### Optum CDM

During the two years leading to the diagnosis of FD, the majority of patients had claims for services from family and preventive medicine (73.1%), radiology (72.1%), and internal medicine (61.2%) specialists. Over 40.0% of patients visited emergency medicine specialists, cardiologists, ophthalmologists, or anesthesiologists (Table 3a). Results were similar for male and females (data not shown). Female patients were more likely to visit emergency medicine specialists than male patients (55.8% vs. 43.3%, respectively).

**Table 3:** Specialist visits among patients with FD in the 2-year baseline period prior to index date

Specialist type	Overall (≥ 1 visit by specialty), *n* (%)	Overall Mean (SD)	Number of visits^a^	
			Female Mean (SD)	Male Mean (SD)
**3a: Optum CDM ** **(N=201)**	n=201	n=201	n=104	n=97
Family and preventive medicine	147 (73.1)	11.6 (17.2)	11.0 (19.0)	12.2 (14.8)
Radiology	145 (72.1)	6.2 (7.8)	7.6 (9.0)	4.5 (5.7)
Internal medicine	123 (61.2)	14.1 (17.5)	15.4 (20.8)	12.6 (13.0)
Surgery	107 (53.2)	4.6 (5.8)	5.1 (6.0)	4.1 (5.5)
Emergency Medicine	100 (49.8)	8.6 (15.5)	9.3 (17.0)	7.7 (13.4)
Ophthalmology	91 (45.3)	3.3 (3.2)	3.8 (4.0)	2.8 (2.0)
Anesthesiology	83 (41.3)	3.6 (5.0)	3.3 (4.3)	3.9 (5.7)
Cardiology	82 (40.8)	8.6 (12.0)	8.9 (13.5)	8.1 (9.9)
Pathology	75 (37.3)	2.2 (2.4)	2.2 (2.1)	2.2 (2.8)
Physical Medicine and Rehabilitation	67 (33.3)	17.2 (31.0)	20.4 (38.7)	12.8 (14.2)
**3b: Komodo RD ** **(N=923)**	n=923	n=923	n=535	n=388
General Practitioner	807 (87.4)	23.7 (30.4)	25.7 (32.2)	20.9 (27.4)
Anesthesiology	238 (25.8)	3.3 (6.6)	3.6 (7.6)	2.9 (4.5)
Radiology	585 (63.4)	5.7 (9)	5.4 (6.6)	6.3 (11.8)
Emergency Medicine	526 (57.0)	5.2 (9.6)	5.8 (10.5)	4.4 (8.1)
Internal medicine	450 (48.8)	17.2 (27.4)	16.6 (27.9)	17.8 (26.9)
Surgery	429 (46.5)	5.2 (7.2)	5.4 (7.8)	5.0 (6.3)
Cardiology	355 (38.5)	14.5 (25.5)	11.0 (20)	18.9 (30.7)
Ophthalmology	332 (36.0)	3.6 (5.9)	3.8 (6.7)	3.3 (4.6)
Pathology	310 (33.6)	4.5 (16.1)	2.9 (4.5)	7.3 (25.8)
Obstetrics/Gynecology	264 (28.6)	8.6 (10.7)	9.3 (11)	2.1 (3.1)
Physical Medicine & Rehabilitation	263 (28.5)	13.6 (23.6)	12.8 (21.6)	14.8 (26.3)

The index date represents the date of first evidence of Fabry disease (i.e., FD diagnosis or treatment)

*CDM,* Clinformatics^®^ Data Mart; *SD,* standard deviation; *RD,* Research Dataset; *SD,* standard deviation

^a^Among patients with at least one visit to the respective specialist

Moreover, a large increase was observed in the proportion of female patients who visited emergency medicine and physical medicine specialists and rehabilitation specialists from year 2 of baseline (33.7% and 19.2%, respectively) to year 1 of baseline (47.1% and 30.8%, respectively). The proportion of male patients that visited pathologists increased from year 2 of baseline (15.5%) to year 1 of baseline (25.8%) (data not shown).

#### Komodo RD

In the two years leading to FD diagnosis, patients had claims for services from the following specialists: general practitioners (87.4%), radiologists (63.4%), emergency medicine specialists (57.0%) and anesthesiologists (25.8%). Over 45.0% of patients visited internal medicine specialists or surgeons **(**Table 3b**)**. Male patients exhibited an increase in the prevalence of visits to radiologists from year 2 of baseline (36.6%) to year 1 of baseline (47.4%) (data not shown).

### Baseline HCRU

#### Optum CDM

In the overall population, nearly all patients (99.0%) had at least one outpatient visit (mean ± SD of 45.7 ± 38.6 and a rate of 22.86 PPPY, data not shown), and 39.8% of patients had at least one ER visit during the two-year baseline period. Additionally, a higher proportion of female patients (43.3%) had at least one ER visit compared to male patients (36.1%). The mean ± SD number of visits among patients with at least one ER visit was 5.3 ± 6.0 for the overall cohort, with a similar mean observed among females and males (5.4 ± 6.8 vs. 5.2 ± 5.0, respectively) (data not shown).

In the Optum dataset, 25.9% of patients experienced all-cause hospitalizations, with females showing nearly double the prevalence of males (33.7% vs. 17.5%) (Table 4). The mean ± SD length of hospital stays in the overall population was 22.5 ± 29.7 days.

**Table 4:** Healthcare resource utilization among patients with FD in the 2-year baseline period prior to index date

HCRU type	Optum CDM			Komodo RD		
	Overall, *N* = 201, *n* (%)	Female, *n* = 104, *n* (%)	Male, *n* = 97, *n* (%)	Overall, *N* = 923, *n* (%)	Female, *n* = 535, *n* (%)	Male, *n* = 388, *n* (%)
Outpatient	199 (99.0)	103 (99.0)	96 (99.0)	911 (98.7)	529 (98.9)	382 (98.5)
Emergency Room	80 (39.8)	45 (43.3)	35 (36.1)	525 (56.9)	308 (57.6)	217 (55.9)
All-cause hospitalization	52 (25.9)	35 (33.7)	17 (17.5)	222 (24.1)	129 (24.1)	93 (24.0)
CVD-related hospitalization^a^	44 (21.9)	27 (26.0)	17 (17.5)	136 (14.7)	66 (12.3)	70 (18.0)
CKD-related hospitalization^a^	11 (5.5)	7 (6.7)	4 (4.1)	43 (4.7)	19 (3.6)	24 (6.2)
Laboratory^b^	105 (52.2)	55 (52.9)	50 (51.5)	398 (43.1)	216 (40.4)	182 (46.9)

The index date represents the date of first evidence of Fabry disease (i.e., FD diagnosis or treatment)

*CKD,* chronic kidney disease; *CDM,* Clinformatics^®^ Data Mart; *CVD,* cardiovascular disease; *HCRU,* healthcare resource utilization; *ICD,* International Classification of Disease; *RD,* Research Dataset

^a^CVD-related and CKD-related hospitalization include a claim with the appropriate ICD-9/ICD-10 code at any position

^b^Unique days with any lab test code

In year 1, the mean ± SD length of hospital stays was 16.7 ± 21.1 days, with female patients having stays approximately four days longer than males (17.8 ± 22.5 days vs. 13.8 ± 17.4 days, respectively), whereas in year 2, the mean ± SD length of hospital stays was 16.1 ± 22.2 days, with male patients having stays approximately two days longer than females (17.1 ± 28.4 days vs. 15.5 ± 18.4). The average hospital stay was slightly longer for females than males (mean: 23.3 vs. 20.8 days, respectively; data not shown).

Furthermore, 21.9% of patients had CVD-related hospitalizations, with a higher prevalence observed among female patients (26.0%) than male patients (17.5%) (Table 4). Similarly, the prevalence of CKD-related hospitalizations was 5.5%, but female patients had slightly higher prevalence than male patients (6.7% vs. 4.1%, respectively). About 52.2% of patients had at least one lab testing day during the baseline period, with nearly equal proportions of female (52.9%) and male (51.5%) patients (Table 4).

#### Komodo RD

The majority of patients with FD (98.7%) had at least one outpatient visit in the baseline period; 56.9% and 24.1% of patients had ER and inpatient visits, respectively. Prevalence of CVD-related and CKD-related hospitalizations was higher among male patients than among female patients (CVD: 18.0% vs. 12.3%) and (CKD: 6.2% vs. 3.6%) (Table 4). Among female patients, a large increase in ER visits was observed from year 2 (36.6%) to year 1 (47.1%) of baseline. Moreover, in the overall population, a large increase in the proportion of patients undergoing any lab testing was observed from year 2 (77.4%) to year 1 (86.7%) of baseline (data not shown).

## Discussion

This retrospective analysis of two large US claims databases provides critical insights into the complex patient journey to FD diagnosis. Our findings revealed a consistent pattern of multisystemic involvement, with high prevalence of cardiovascular (56–73%), neurologic (59–64%), and gastrointestinal (46–51%) manifestations in the two years preceding diagnosis. This pattern aligns with the known pathophysiology of FD as a progressive, multi-organ disorder [8], including studies in Germany and Japan [18, 19], but – importantly – our study quantifies the substantial pre-diagnostic burden across multiple healthcare systems and patient populations.

While the prevalence of specific comorbidities was markedly higher in our FD population compared to general US population estimates (e.g. arrhythmia in 25–30% of patients with FD versus 1–2% in the general population [20, 21], and chronic kidney disease prevalence nearly double that of the adult US population [22], these findings should be interpreted with caution. Several factors may contribute to potential overestimation of comorbidity rates in our study. First, the claims-based nature of our data might inflate prevalence estimates due to coding practices, where multiple diagnostic codes may be entered during the diagnostic workup process. Second, our study population was notably older than typical FD cohorts, particularly in the Optum CDM database (mean age 56.0 years), which could lead to higher comorbidity rates due to age-related conditions. Third, patients with more severe disease or multiple comorbidities may be more likely to seek medical care and, thus, be captured in claims databases. Despite these potential sources of overestimation, the consistent pattern of elevated comorbidity rates across both databases and their alignment with known FD manifestations suggest that these findings reflect genuine disease burden, even if absolute rates may be somewhat inflated.

The demographic composition of our study population, predominantly older patients, reflects both the nature of the databases and our inclusion criteria. While this may limit our ability to characterize the journey of classic male patients, who are often diagnosed earlier in life [1, 23], it provides valuable insights into the more attenuated phenotypes (classic females and later-onset patients), who often face prolonged diagnostic odysseys due to non-specific symptom presentation [24].

Despite the presentation of various symptoms, patients with FD often remain undiagnosed for many years [17]. Within the two-year observation window prior to FD diagnosis, we observed frequent specialist visits, ER visits, and inpatient stays. This high HCRU suggests inadequate diagnosis and disease awareness, leading patients to seek care from various medical specialties and potentially undergo multiple exploratory assessments without addressing the root cause.

The most common provider specialties associated with patients’ first observed FD diagnosis codes were general practitioners, internal medicine specialists, or medical geneticists. This finding underscores the critical role of primary care and internal medicine in recognizing and initiating the diagnostic process for FD. The diverse clinical presentation of FD poses challenges for accurate and timely diagnosis, making it imperative for specialists to be vigilant for potential FD red flag indicators including renal, cardiovascular, or neurologic involvement [8]. Early recognition by primary care providers, coupled with appropriate specialist referrals, could significantly reduce the overall disease burden through earlier diagnosis and timely treatment initiation.

In terms of specific diagnostic opportunities, FD has a notable prevalence among patients with end-stage renal disease (ESRD), with rates of 0.21% in males and 0.15% in females undergoing hemodialysis screening, and 0.25% in males among renal transplant candidates. In patients with left ventricular hypertrophy (LVH), the prevalence is even higher – 0.94% in males and 0.90% in females. These findings emphasize the importance of targeted screening in these high-risk populations [25].

Early identification of FD is critical, as timely initiation of disease-specific therapies can significantly improve clinical outcomes. In the context of cardiomyopathies, phenotype-directed multigene panels are now recommended as a first-line diagnostic strategy, owing to their favorable cost-effectiveness and high diagnostic yield [26]. Similarly, there is a growing consensus supporting the use of next-generation sequencing with multigene panels in patients with chronic kidney disease (CKD) to enhance diagnostic precision and enable personalized medical management [27]. Given these developments, it is essential to ensure that the *GLA* gene is consistently included in multigene panels used for the evaluation of CKD and cardiomyopathy, to avoid missed or delayed diagnoses of FD.

Based on our findings and prior literature, we propose the following actionable recommendations to improve FD diagnosis:

1. Develop multidisciplinary red-flag alerts for electronic health record systems to identify patients with unexplained multisystemic symptoms consistent with FD.
2. Enhance education and awareness programs for healthcare providers, particularly in primary care, internal medicine, cardiology, and neurology, about the varied presentations of FD and the importance of early diagnosis.
3. Encourage consolidation of patient history across specialties to better recognize patterns suggestive of rare diseases like FD.
4. Consider testing for FD in patients with unexplained chronic kidney disease, unexplained left ventricular hypertrophy or neuropathic pain.

Our study has several important limitations that should be considered when interpreting the results:

- Reliance on administrative claims data for identifying FD cases, without the ability to confirm diagnoses through genetic or enzymatic validation. While we attempted to mitigate potential misclassification by requiring either multiple diagnosis codes or evidence of FD-specific treatment, this approach may include patients with provisional or incorrect FD diagnoses and may miss patients with non-classical variants or atypical presentations. These limitations are particularly relevant for a heterogeneous condition like FD, where phenotypic expression varies widely, especially between males and females and across different genetic variants.
- Limited information on specific reasons for hospitalization beyond CVD- and CKD-related admissions, despite the observed extended hospital stays.
- Exclusion of Medicaid patients in the Optum CDM dataset, potentially underrepresenting early-onset cases, particularly classic male patients who are typically diagnosed earlier in life.
- Possibility for some degree of patient overlap between the two considered claims databases. Nonetheless, the impact on our finding is likely minimal for several reasons: (1) the databases cover different time periods (Optum CDM: March 2012 to March 2022; Komodo RD: January 2018 to September 2022), (2) there are differences in the patient populations represented in the two databases: Optum CDM captures only individuals who are covered by commercial insurance or Medicare through a single payer, whereas Komodo RD includes patients covered by multiple commercial and Medicare payers as well as Medicaid, (3) we required two years of continuous enrollment prior to the first recorded Fabry diagnosis to increase the likelihood that we captured patients’ incident diagnosis, (4) our analyses were conducted independently for each database, and results are reported separately.
- Use of ICD codes without confirmation of clinical diagnoses, although this is common in claims data analyses.
- Potential misclassification of patients as newly diagnosed due to limited observability in the databases.
- Lack of information about services not resulting in insurance claims, such as over-the-counter medications.
- Potential coding errors, omissions, and lack of specific billing codes for some conditions in administrative claims data.
- Impact of the COVID-19 pandemic on healthcare utilization during the study period, potentially affecting the patient journey to FD diagnosis.

Future studies should aim to address these limitations by combining claims data with clinical records, genetic testing results, or biomarker data. This would provide more definitive patient identification and allow for stratification by disease subtype and severity. Additionally, prospective studies tracking patients from initial symptom onset through diagnosis could offer more precise insights into the diagnostic journey and identify critical points for intervention.

Our study provides a comprehensive overview of the pre-diagnostic health state of patients with FD, highlighting the multisystemic nature of the disease and the challenges in its timely diagnosis. By increasing awareness of the diverse presentations of FD and implementing targeted screening strategies, we can work towards earlier diagnosis and improved outcomes for patients with this rare but significant disorder.

## Conclusions

This retrospective, observational study of patients with FD represented in two US claims databases (Optum CDM and Komodo RD) demonstrated high burden of cardiovascular, neurologic, and gastrointestinal symptoms and comorbidities, with associated medication use prior to diagnosis. The consistency of the results obtained from the two databases reinforces these findings. Recognition of these patterns in patients with FD may lead to shorter time to diagnosis and better disease management, resulting in improved outcomes (e.g., prevention of organ damage, delayed clinical events, and improved quality of life).

## Supplementary Information

Supplementary Material 1.

## Data Availability

The datasets used and/or analyzed during the current study are available from the corresponding author upon reasonable request and may be subject to a data sharing agreement.

## Abbreviations

α-Gal A: α-Galactosidase A
CKD: Chronic kidney disease
CVD: Cardiovascular disease
ER: Emergency room
FD: Fabry disease
FDA: Food and Drug Administration
GL-3: Globotriaosylceramide
HIPAA: Health Insurance Portability and Accountability Act
ICD-9: International Classification of Diseases-9
ICD-10: International Classification of Diseases-10
IQR: Interquartile Range
HCRU: Healthcare resource utilization
Komodo RD: Komodo Research Dataset
Optum CDM: Optum Clinformatics^®^ Data Mart
PPPY: Per patient per year
SD: Standard deviation
US: United States
