# Supplementary material for: Patient journey to Fabry disease diagnosis in the United States: an observational retrospective analysis of two United States claims databases
Source: Orphanet J Rare Dis. 2025 Nov 27;20:613. doi: 10.1186/s13023-025-04041-3 (PMC12659568; doi:10.1186/s13023-025-04041-3)
Supplement: Supplementary file 1 — Supplementary Material 1. [file 13023_2025_4041_MOESM1_ESM.docx]

# Supplementary materials

**Supplementary Table 1: Specialist visits for newly diagnosed patients with FD prior to index date^*^ in Optum CDM**

|  | **Year 2 of baseline** | | | **Year 1 of baseline** | | |
| --- | --- | --- | --- | --- | --- | --- |
|  | **Overall (≥1 visit by specialty),**  **N=201,**  **n (%)** | **Female**  **n=104,** **Mean (SD)** | **Male**  **n=97, Mean (SD)** | **Overall (≥1 visit by specialty),**  **N=201,**  **n (%)** | **Female**  **n=104, Mean (SD)** | **Male**  **n=97, Mean (SD)** |
| Anesthesiology | 50 (24.9) | 2.5 (3.0) | 2.5 (3.0) | 51 (25.4) | 2.9 (2.9) | 4.0 (4.9) |
| Behavioral care | 29 (14.4) | 6.2 (13.6) | 4.3 (3.7) | 34 (16.9) | 5.1 (7.9) | 3.6 (2.4) |
| Cardiology | 57 (28.4) | 4.2 (6.2) | 5.9 (7.6) | 63 (31.3) | 8.2 (10.2) | 4.8 (3.7) |
| Dermatology | 34 (16.9) | 1.9 (1.0) | 2.7 (3.6) | 41 (20.4) | 1.9 (1.7) | 2.4 (1.6) |
| Emergency Medicine | 67 (33.3) | 6.5 (9.9) | 4.7 (8.8) | 83 (41.3) | 6.4 (9.7) | 5.1 (7.3) |
| Family & Preventive Medicine | 125 (62.2) | 5.7 (10.1) | 6.8 (8.4) | 130 (64.7) | 7.1 (11.3) | 7.1 (8.3) |
| Gastroenterology | 33 (16.4) | 1.9 (1.3) | 2.2 (1.4) | 27 (13.4) | 2 (1.8) | 2 (1.2) |
| Internal medicine | 97 (48.3) | 8.2 (11.7) | 8.0 (8.1) | 106 (52.7) | 10 (12.6) | 7.6 (7.9) |
| Neurology | 23 (11.4) | 4.9 (7.3) | 1.9 (0.6) | 27 (13.4) | 3.6 (3.0) | 1.9 (1.3) |
| Obstetrics & Gynecology | 38 (18.9) | 2.8 (4.2) | 2.0 (1.1) | 40 (19.9) | 3.3 (3.6) | 2.4 (2.3) |
| Ophthalmology | 70 (34.8) | 2.1 (2.2) | 1.8 (1.3) | 70 (34.8) | 2.9 (3.6) | 1.8 (1.4) |
| Other | 35 (17.4) | 7.5 (13.4) | 5.2 (8.0) | 46 (22.9) | 8.4 (11.1) | 2.2 (1.9) |
| Pathology | 43 (21.4) | 1.7 (1.1) | 2.3 (3.8) | 56 (27.9) | 1.6 (1.3) | 1.4 (0.9) |
| Pediatrics | 17 (8.5) | 4.5 (7.3) | 2.9 (1.9) | 22 (10.9) | 3.9 (5.8) | 3.0 (4.7) |
| Physical Medicine and Rehabilitation | 40 (19.9) | 13.9 (19.3) | 7.6 (5.2) | 52 (25.9) | 16.2 (36.6) | 10.4 (11.2) |
| Podiatry | 16 (8.0) | 3.6 (2.7) | 4.0 (2.0) | 21 (10.4) | 6.7 (9.6) | 2.5 (1.4) |
| Radiology | 117 (58.2) | 3.9 (4.4) | 2.8 (4.5) | 112 (55.7) | 5.3 (6.9) | 3.2 (2.9) |
| Surgery | 70 (34.8) | 3.8 (4.7) | 3.3 (4.6) | 73 (36.3) | 3.6 (4.0) | 3.2 (2.6) |
| Urology | 23 (11.4) | 8.0 (8.5) | 1.9 (1.4) | 19 (9.5) | 2.6 (1.8) | 2.2 (1.5) |
| Urgent care | 22 (10.9) | 1.6 (1.4) | 1.6 (0.9) | 15 (7.5) | 2.3 (2.9) | 1.2 (0.4) |

CDM, Clinformatics^®^ Data Mart; FD, Fabry disease; SD, standard deviation.
*Among patients with at least one visit to the respective specialist. A cut-off of ≥10% has been applied to the overall column in this table.

**Supplementary Table 2: Specialist visits for newly diagnosed patients with FD prior to index date^*^ in Komodo RD**

| **Specialist** | **Year 2 of baseline** | | | **Year 1 of baseline** | | |
| --- | --- | --- | --- | --- | --- | --- |
|  | **Overall (≥1 visit by specialty),**  **N=923,**  **n%** | **Female**  **n=535, Mean (SD)** | **Male**  **n=388,  Mean (SD)** | **Overall (≥1 visit by specialty),**  **N=923,**  **n (%)** | **Female**  **n=535, Mean (SD)** | **Male**  **n=388, Mean (SD)** |
| Anesthesiology | 120 (13.0) | 2.7 (5.1) | 2.3 (2.6) | 173 (18.7) | 3.2 (5.2) | 2.3 (2.6) |
| Behavioral care | 127 (13.8) | 9.9 (9.8) | 12.4 (19.6) | 162 (17.6) | 10.5 (11.7) | 15.6 (43.5) |
| Cardiology | 222 (24.1) | 7.6 (9.5) | 10.5 (12.9) | 301 (32.6) | 7.7 (13.7) | 13.9 (23.9) |
| Dermatology | 98 (10.6) | 3.4 (3.8) | 3.5 (3.7) | 121 (13.1) | 3.2 (4.8) | 2.8 (2.7) |
| Emergency medicine | 332 (36) | 4.0 (7.0) | 3.0 (5.4) | 405 (43.9) | 4.1 (7.0) | 3.5 (5.5) |
| Gastroenterology | 117 (12.7) | 3.8 (3.9) | 4.8 (6.7) | 135 (14.6) | 4.2 (5.4) | 5.7 (7.3) |
| General Practitioner | 684 (74.1) | 12.9 (16.2) | 11.5 (14.5) | 736 (79.7) | 15.6 (18.4) | 13.0 (15.8) |
| Hematology & oncology | 59 (6.4) | 6.3 (8.2) | 15.6 (26.3) | 94 (10.2) | 5.7 (6.8) | 7.3 (14.6) |
| Internal medicine | 322 (34.9) | 10.4 (15.4) | 10.2 (14.4) | 370 (40.1) | 11.5 (17.3) | 12.4 (17.0) |
| Neurology | 99 (10.7) | 8.5 (11.6) | 6.4 (9.5) | 136 (14.7) | 9.3 (11.9) | 4.8 (7.9) |
| Nephrology | 59 (6.4) | 15.7 (26.1) | 16.3 (39.9) | 94 (10.2) | 11.1 (17.9) | 15.3 (21.5) |
| Nursing | 88 (9.5) | 1.3 (0.6) | 1.5 (1.2) | 104 (11.3) | 1.5 (1.8) | 1.8 (1.5) |
| Other | 208 (22.5) | 4.1 (8.9) | 4.9 (9.2) | 229 (24.8) | 4.3 (8.7) | 5.5 (9.1) |
| Obstetrics & gynecology | 180 (19.5) | 6.4 (7.1) | 1.5 (1.1) | 206 (22.3) | 6.0 (7.4) | 2.4 (3.2) |
| Ophthalmology | 218 (23.6) | 2.8 (4.1) | 2.4 (2.7) | 233 (25.2) | 2.8 (4.3) | 2.4 (2.9) |
| Pathology | 166 (18.0) | 2.4 (3.6) | 5.9 (15.0) | 234 (25.4) | 2.2 (2.5) | 5.4 (17.2) |
| Pediatrics | 177 (19.2) | 4.7 (6.2) | 6.1 (7.0) | 180 (19.5) | 5.1 (6.3) | 5.9 (7.8) |
| Physical medicine and rehabilitation | 172 (18.6) | 9.3 (14.3) | 11.4 (16.2) | 200 (21.7) | 8.8 (12.8) | 9.8 (18.0) |
| Pulmonary medicine | 78 (8.5) | 5.2 (7.0) | 6.2 (7.3) | 96 (10.4) | 6.1 (8.4) | 6.2 (7) |
| Radiology | 394 (42.7) | 3.3 (3.5) | 3.4 (4.1) | 482 (52.2) | 3.7 (4.1) | 5.2 (10.8) |
| Surgery | 263 (28.5) | 3.8 (5.0) | 3.8 (4.2) | 328 (35.5) | 3.9 (4.6) | 3.7 (4.2) |

FD, Fabry disease; RD, Research Dataset; SD, standard deviation.
*Among patients with at least one visit to the respective specialist. A cut-off of ≥10% has been applied to the overall column in this table.
